# Computational Analyses of Synergism in Small Molecular Network Motifs

**DOI:** 10.1371/journal.pcbi.1003524

**Published:** 2014-03-20

**Authors:** Yili Zhang, Paul Smolen, Douglas A. Baxter, John H. Byrne

**Affiliations:** Department of Neurobiology and Anatomy, The University of Texas Medical School at Houston, Houston, Texas, United States of America; University of California San Diego, United States of America

## Abstract

Cellular functions and responses to stimuli are controlled by complex regulatory networks that comprise a large diversity of molecular components and their interactions. However, achieving an intuitive understanding of the dynamical properties and responses to stimuli of these networks is hampered by their large scale and complexity. To address this issue, analyses of regulatory networks often focus on reduced models that depict distinct, reoccurring connectivity patterns referred to as motifs. Previous modeling studies have begun to characterize the dynamics of small motifs, and to describe ways in which variations in parameters affect their responses to stimuli. The present study investigates how variations in pairs of parameters affect responses in a series of ten common network motifs, identifying concurrent variations that act synergistically (or antagonistically) to alter the responses of the motifs to stimuli. Synergism (or antagonism) was quantified using degrees of nonlinear blending and additive synergism. Simulations identified concurrent variations that maximized synergism, and examined the ways in which it was affected by stimulus protocols and the architecture of a motif. Only a subset of architectures exhibited synergism following paired changes in parameters. The approach was then applied to a model describing interlocked feedback loops governing the synthesis of the CREB1 and CREB2 transcription factors. The effects of motifs on synergism for this biologically realistic model were consistent with those for the abstract models of single motifs. These results have implications for the rational design of combination drug therapies with the potential for synergistic interactions.

## Author Summary

Cellular responses to stimuli are controlled by complex regulatory networks that comprise many molecular components. Understanding such networks is critical for understanding normal cellular functions and pathological conditions. Because the complexity of these networks often precludes intuitive insights, a useful approach is to study mathematical models of small network motifs having reduced complexity yet consisting of key regulatory components of the more complex networks. Computational studies have analyzed the behavior of small motifs, and have begun to describe the ways in which variations in parameters affect their functional properties. Here, we investigated how variations in pairs of parameters act synergistically (or antagonistically) to alter responses of ten common network motifs. Simulations identified parameter variations that maximized synergism, and examined the ways in which synergism was affected by stimulus protocols and motif architecture. The results have implications for the rational design of combination drug therapies where a goal is to identify drugs that when administered together have a greater effect than would be predicted by simple addition of single-drug effects (*i.e.*, super-additive effects), thereby allowing for lower drug doses, minimizing undesirable effects.

## Introduction

Cellular functions are regulated by complex biochemical networks that incorporate large numbers of diverse molecular components and their interactions. The large scale and complexity of these regulatory networks impedes achieving an intuitive understanding of their overall function and responses to stimuli and/or drugs. Consequently, when analyzing a complex system, it is often useful to develop and analyze reduced models that capture the key dynamical properties of the system. In analyses of biochemical networks, these reduced models are referred to as motifs [1]. Motifs depict distinct connectivity patterns that occur more frequently in a given network than in random networks of the same size. Motifs can be comprised of as few as three molecules (referred to as nodes or vertices) and their interactions (referred to as edges). Motifs are present in gene regulatory networks, protein-protein interactions, and metabolic networks of species as diverse as bacteria [1]–[2], yeast [2]–[3], and humans [2], [4]–[12]. Structurally distinct motifs appear to manifest specific dynamical features [10], [13]–[17] and modeling studies describe how the responses of distinct motifs and the robustness of these responses vary with parameters [18]–[20]. These studies are beginning to elucidate ways in which motif dynamics contribute to the functions and response properties of larger, more complex regulatory networks. Moreover, as is investigated here, small network motifs can be used to examine the ways in which combinations of parameter changes act synergistically (or antagonistically) to alter the response to stimuli. This later strategy may ultimately help guide the development of drug combination therapies that target disease-related dysfunction of a network motif.

**Figure 1:**
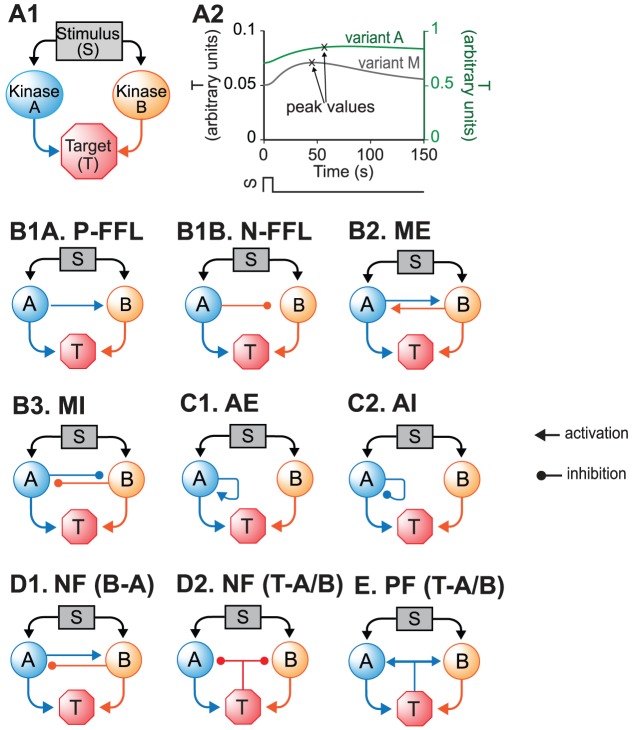
Canonical model and variations. (**A1**) The canonical model describes the convergence of two signaling pathways (elements A and B) onto target (T). A single stimulus (*S*) activates both A and B. Then, A and B positively regulate the activation of T. (**A2**) For the canonical model, the stimulus-response curves are transient in both model variants M and A (S = 1). (**B–E**) Various network motifs derived from the canonical model. (**B1**) Two types of coherent feed-forward loop (FFL). (**B2**) Positive feed-back loop between A and B (*i.e.*, mutual excitation, ME). (**B3**) Negative feed-back loop between A and B (*i.e.*, mutual inhibition, MI). (**C1**) Positive auto-regulation loop of A (*i.e.*, auto-excitation, AE). (**C2**) Negative auto-regulation loop of A (*i.e.*, auto-inhibition, AI). (**D1**) Negative feedback between A and B (NF (B-A)). (**D2**) Negative feedback from T to A and B (NF (T-A/B)). (**E**) Positive feedback from T to A and B (PF (T-A/B)).

Here, models of ten three-node motifs (Fig. 1) were developed and synergistic interactions within these motifs were investigated. These motifs are ubiquitous and are included within gene and protein networks that are associated with specific diseases [1]–[2], [4]–[6], [9], [11], [13], [15], [21]–[24]. The mechanisms of disease are usually associated with large networks of molecular pathways. However, in many clinical studies in which combination drug therapy is used for treatment of diseases, two-drug combinations are considered [25]. Therefore, for models of simple motifs or of complex pathways, simulation of concurrent paired parameter changes is of value for understanding the synergistic or antagonistic properties of many current or possible combination therapies. In the simulations, pairs of parameters were simultaneously varied, and the extent to which these ten motifs manifest synergism (or antagonism) was examined. First, a canonical model was developed. This motif constitutes a minimal representation of two convergent pathways. Element A and element B both respond to a common stimulus (S), and converge to activate a common target (T) (Fig. 1A1). Activation (*e.g.*, increased phosphorylation, or enhanced synthesis) of T was assumed to be the output of the motif, and the target for examining the effects of combinations of parameter variations. Variations of parameter pairs in elements A and B represent combination therapies in which two drugs target two different sites of the same pathway or two pathways converging at a downstream process. Such convergence is commonly used in designing therapies. For example, Paclitaxel synergizes with Tubacin in enhancing tubulin acetylation, with the former directly increasing acetylation and the later decreasing the deacetylation of α-tubulin [25]. Aplidin and Cytarabine are synergistic in killing cancer cells because they induce apoptosis via two convergent signaling cascades [25]. In this study, synergism (or antagonism) was quantified using degrees of nonlinear blending and additive synergism (see Model Development). Then the canonical motif was modified to generate a set of similar three-node motifs that incorporated different patterns of interaction among the nodes. These interactions included a variety of positive and negative feedback loops, and autoregulatory loops. These motifs were found to greatly modify the existence and amount of synergism. For specific parameter pairs, only a subset of motif architectures exhibited synergism.

To substantiate these conclusions, the approach was applied to a model representing interlocked feedback loops that govern the synthesis of two transcription factors, cAMP-response element binding proteins (CREBs), specifically CREB1 and CREB2 [26]. CREB1 is a transcription activator and CREB2 is a transcription repressor. CREB1 and CREB2 regulate their own expression, *via* binding to the CRE elements in or near their genes. The feedback loops involving CREB1 and CREB2 modulate long-term memory [27]. Three of the network motifs that were simulated are included in this model: a positive auto-regulatory loop governing CREB1 synthesis; a negative auto-regulatory loop governing CREB2 synthesis; and negative feedback in which CREB2 inhibits the synthesis of CREB1. The effects of these motifs on synergism in this more biologically realistic model were consistent with the results from the more abstract three-node models.

## Methods

### A canonical model of converging pathways that regulate a downstream target

Elements A and B converge onto a target (T) (Fig. 1A1). Both A and B are activated by stimulus *S*. The activities of A and B are dynamic variables that follow first-order ordinary differential equations (ODEs). *k_basal_A_* and *k_basal_B_* are basal activation rates of *A* and *B*. The deactivation of *A* and *B* follows Michaelis–Menten kinetics. These assumptions yield the following ODEs:
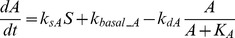
(1)

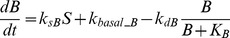
(2)


Two variants of this simple converging model were analyzed. In Variant M, the activation rate of *T* is proportional to the product of the effects of *A* and *B*. Deactivation of T follows Michaelis-Menten kinetics. The following ODE for *T* results:
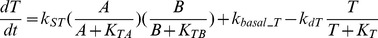
(3)


In Variant A, the activation rate of *T* is proportional to the sum of the effects of *A* and *B*, yielding the following ODE:
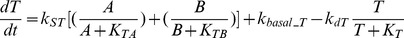
(4)


Concentrations are non-dimensional. Standard parameter values are used in all simulations unless noted. These values are:

*K_sA_* = 0.1, *k_basal_A_* = 0.1, *k_dA_* = 0.2, *K_A_* = 1, *K_sB_* = 0.1, *k_basal_B_* = 0.1, *k_dB_* = 0.2, *K_B_* = 1, *k_basal_T_* = 0.0001, *k_ST_* = 0.01, *K_TA_* = 2.5, *K_TB_* = 2.5, *k_dT_* = 0.01, *K_T_* = 0.5.

The parameters were adjusted by trial and error so that the dynamics of *A*, *B* and *T* display properties of common biochemical responses; *e.g.*, 1) the activation of *A* and *B* was rapid, whereas their deactivation was relatively slow; 2) the peak level of *T* was well below saturation when stimuli were weak; and 3) the basal activation rate of *T*, *k_basal_T_*, was much smaller than the activation rate induced by *A* and *B*, *k_ST_*. For simplicity, the strengths of both pathways were initially balanced. Therefore, the parameters of dynamics of *A* and *B* shared the same values. The extent to which the synergism is dependent on these values is discussed in the Results.

### Expanding the canonical model to represent additional three-node motifs

In the three-node model, it was assumed that 1) both A and B have excitatory effects on T; and 2) the standard parameter values governing A and B dynamics are identical. There are then nine biochemical variations of the canonical motif that involve adding a single feedback or feedforward interaction, or autoregulation, involving A or B. These nine motifs were represented by extensions of the canonical model. For simplicity and consistency, these extensions were all constructed by adding regulation, within the motif, of a given pair of parameters, *k_dA_* and *k_dB_*. After construction, synergism was examined with paired parameter variations for each of these motifs.

#### Coherent, positive feed-forward loop (FFL) (Fig. 1B1)

In FFLs, A regulates B, and both regulate the target [28]. The FFL has three interactions (A to B, A to T, and B to T). Each of these interactions can be either positive (activation) or negative (repression). Thus, eight variants are possible for the FFL. Four of these variants are referred to as *coherent* because the sign of direct regulation from A to T is the same as the overall sign of the indirect regulatory pathway (A to B to T). Four other variants are referred to as *incoherent* because the signs of the direct and indirect pathways are opposite. The present study focused on one coherent FFL variant (P-FFL), which is frequently observed in signaling pathways [29] (Fig. 1B1A), and one incoherent FFL variant (N-FFL) (Fig. 1B1B). To generate the P-FFL, *k_dB_* was assumed to decrease with increasing *A*.
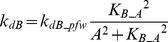
(5)where *k_dB_pfw_* = 0.22, *K_B_A_* = 3.

To generate the N-FFL, *k_dB_* was assumed to increases with increasing *A*.
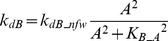
(6)where *k_dB_nfw_* = 0.4, *K_B_A_* = 1.

#### Crosstalk (either a positive-feedback, mutual excitation loop, Fig. 1B2, or a mutual inhibition loop, Fig. 1B3) between the two pathways

Two types of crosstalk between elements A and B were examined. To add mutual excitation, the deactivation rates of both elements, *k_dA_* and *k_dB_*, were assumed to depend on the levels of *B* and *A* respectively. *k_dA_* decreases with increasing *B*, whereas *k_dB_* decreases with increasing *A*. Strong positive feedback might induce a bistable switch [17], which would, due to saturation, tend to prevent any synergistic effect of simulated parameter variations. Hence a relatively weak mutual excitation loop was implemented.
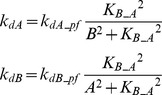
(7)where *k_dA_pf_* = 0.22, *k_dB_pf_* = 0.22, *K_B_A_* = 3.

To add mutual inhibition to the canonical model, *k_dA_* and *k_dB_* were assumed to increase with increasing *B* and *A*, respectively. Mutual inhibition constitutes a form of positive feedback, and strong feedback could induce bistability. Hence a relatively weak mutual inhibition loop was implemented.
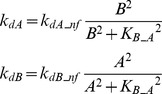
(8)where *k_dA_nf_* = 0.4, *k_dB_nf_* = 0.4, *K_B_A_* = 1.

#### Auto-regulatory loop(s) (Figs. 1C1 and 1C2)

In an auto-regulatory loop, a node directly influences its own function. Common examples of auto-regulation include autophosphorylation of kinases and transcriptional regulation [27], [30]–[31]. To add positive auto-regulation, *k_dA_* was assumed to decrease with increasing *A*.
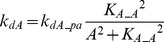
(9)where *k_dA_pa_* = 0.22, *K_A_A_* = 3

To add negative auto-regulation, *k_dA_* was assumed to increase with increasing *A*.
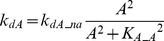
(10)where *k_dA_na_* = 0.4, *K_A_A_* = 1.

#### Negative feedback between A and B (Fig. 1D1) or from T to A and B (Fig. 1D2)

We considered a negative feedback loop in which activation of A inhibits de-activation of B, with activation of B then accelerating de-activation of A. *k_dA_* was assumed to increase with increasing *B* and *k_dB_* was assumed to decrease with increasing *A*.
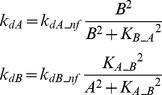
(11)where *k_dA_nf_* = 0.4, *k_dB_nf_* = 0.22, *K_B_A_* = 1, *K_A_B_* = 3.

A negative feedback loop was also implemented in which activation of the target accelerates de-activation of the upstream elements A and B. *k_dA_* and *k_dB_* were assumed to increase with increasing *T*.
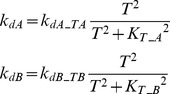
(12)where *k_dA_TA_* = 0.4, *k_dB_TB_* = 0.4, *K_T_A_* = 0.05, *K_T_B_* = 0.05.

#### Positive feedback from T to A and B (Fig. 1E)

Here *k_dA_* and *k_dB_* were assumed to decrease with increasing *T*, inhibiting de-activation of the upstream elements A and B.
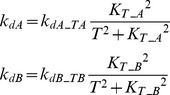
(13)where *k_dA_TA_* = 0.22, *k_dB_TB_* = 0.22, *K_T_A_* = 0.15, *K_T_B_* = 0.15.

### The model of Song et al. [26]

CREB1 is assumed to bind to cAMP response elements (CREs) near the promoters of both the *creb1* and *creb2* genes, activating expression of both genes. CREB2 is assumed to bind competitively to the same CREs and to repress transcription of both genes. In this minimal model, differential equations for mRNAs are not included. Thus the model consists of two ODEs, for the levels of CREB1 and CREB2, with gene regulation represented by activation and repression of CREB1 and CREB2 formation.
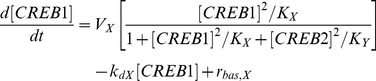
(14)

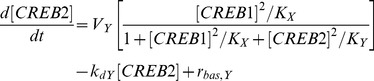
(15)Concentrations are non-dimensional. Standard parameter values from Song et al. [26] are used. These values are:

*V_x_* = 0.4 min^−1^, *V_y_* = 0.01 min^−1^, *K_x_* = 5, *K_y_* = 10, *k_dx_* = 0.04 min^−1^, *k_dy_* = 0.01 min^−1^, *r_bas,x_* = 0.003 min^−1^, and *r_bas,y_* = 0.002 min^−1^

The parameters involved in the positive auto-regulatory loop in which CREB1 enhances its own synthesis are *V_x_* (the maximum induced synthesis rate of CREB1) and *K_x_* (the dissociation constant of CREB1 from a CRE). The parameters involved in the negative auto-regulatory loop in which CREB2 represses its own synthesis are *V_y_* (the maximum induced synthesis rate of CREB2) and *K_y_* (the dissociation constant of CREB2 from a CRE). All four parameters are involved in the negative feedback loop in which CREB2 represses the synthesis of CREB1, because in this loop, CREB1 first activates CREB2 synthesis (parameters *V_y_*, *K_x_*) and CREB2 then represses CREB1 synthesis (parameters *V_x_*, *K_y_*).

### Quantification of nonlinear blending synergism and additive synergism

Measures to assess synergistic drug actions are diverse and include additive synergism [32]–[34], Bliss independence [32], [35]–[37], the Chou-Talalay Combination Index [37], the isobolographic approach [32], Loewe additivity [33]–[35], and nonlinear blending [38], but there is no agreement on which is preferable. Given the diversity of methods for measuring synergism, it is useful to adopt more than one method in studies of combination drug treatments. In the present study nonlinear blending synergism and additive synergism were selected to assess the effects of combinations of parameter variations on the output of the motifs. These methods were chosen because: 1) Additive synergism is a straightforward way to calculate synergism and can be easily conceptualized, and 2) Nonlinear blending allows for synergism to be assessed by considering the shape of a curve constructed over a range of concurrent drug dosages, as opposed to assessing at a single dose. In this way, nonlinear blending is closely related to several of the more complex methods for calculating synergism, such as isobolograms.

**Figure 2:**
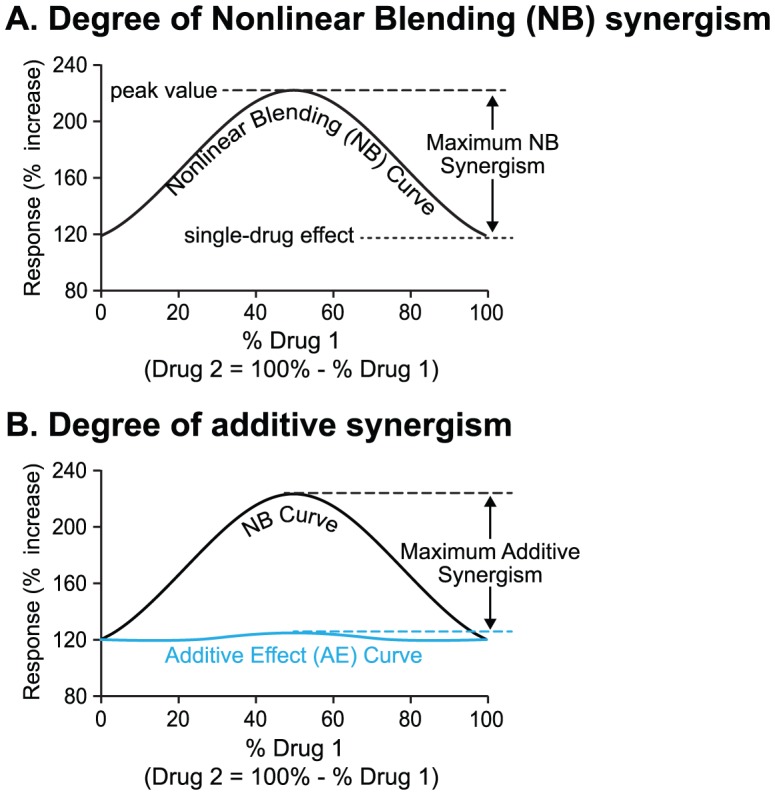
A schematic of NB synergism (A) and additive synergism (B). Response for AE curve = effect of drug 1 alone+effect of drug 2 alone. The maximal difference between peak value and endpoints of NB curves determines the maximum NB synergism. The maximal difference between NB and AE curves determines maximum additive synergism.

In nonlinear blending [38], a fixed total amount of drug 1 is selected, which gives a substantial, but not saturated, response. Then the response is quantified for mixtures of drugs 1 and 2, holding the total drug amount the same as with drug 1 alone, but varying the percentage of drug 1 in the mixture from 0 to 100%. The resulting dose-effect curve, with percentage of drug 1 on the *x* axis, will be concave up if antagonism is present between the drugs and concave down if synergism is present. Weak nonlinear blending synergism is present if the curve is concave down, but still has its maximum at an end point [38]. Weak nonlinear blending may not be useful, however, because the maximal response is still obtained at one endpoint, using only one parameter change. However a stronger, more useful form of synergism may be seen, termed strong nonlinear blending synergism [38]. In this case, the curve is concave down with the maximum response at a point removed from either end point (Fig. 2A). Thus, for a given total drug amount, the best response is obtained for a mixture of drugs 1 and 2.

For each nonlinear blending (NB) curve, a corresponding additive effect (AE) curve can be constructed. The same combination of drugs is used as for the NB curve. However, the additive effect is simply calculated as the sum of the response from with the altered value of drug 1 alone and the response with drug 2 alone (Fig. 2B). Additive synergism is then present if the response to the combination of drugs 1 and 2, shown by the NB curve, is greater than the sum of the responses to the individual drugs, shown by the AE curve.

In the simulations, the effects of pairs of drugs were mimicked by varying pairs of parameters. In the canonical model, 14 parameters in Eq. 1–4 determine the dynamics of *A*, *B* and *T*. The peak level of *T* was considered to represent the response to the stimulus *S* (Fig. 1A2). The peak level of *T* in the absence of parameter changes was regarded as the control peak level. The percentage increase of the peak level with parameter changes over the control peak level was taken as the simulated response to the parameter changes. Individual parameters were varied in the direction that increased the peak level of *T*. For 14 parameters, 91 distinct combinations of two parameters are possible. To simulate a reasonable range of drug effect strengths, each individual parameter was varied within the range bounded by 90% changes of its standard value, either increased (0%–+90%) or decreased (0%–−90%), according to which direction increased the peak level of *T*. When two parameters were modified simultaneously, the sum of absolute values of individual parameter percent changes was maintained at 90%. The value of 90% was chosen so that the maximal degree of reduction, or inhibition, of biochemical processes governed by these parameters – synthesis, degradation, or activation/deactivation of A, B, or T – was 90%. For example, *k_dA_*/*k_ST_* is one pair that was selected. For simulations with this pair, *k_dA_* was decreased, because a decrease in the degradation rate of *A* tends to increase the peak level of the target *T*, and *k_ST_* was increased, because an increase in the maximum induced synthesis rate of *T* also increases the peak level of *T*. In a series of combined parameter changes, the decrease in *k_dA_* varied from 0% to 90% of its standard value, and the increase in *k_ST_* concurrently varied from 90% to 0% of its standard value, such that |% decrease in *k_dA_*|+|% increase in *k_ST_*| = 90%. To simulate dose-effect curves for individual parameters, as well as to construct NB curves, 30 points were used, evenly spaced between 0 and 90% parameter variations from standard values, evenly spaced between 0 and 90% parameter variations from standard values.

Synergism was quantified by defining the degrees of NB and additive synergism. The left end point of an NB curve corresponds to a 90% change in parameter 2 (*i.e.*, 0% change in parameter 1). The right end point corresponds to a 90% change of parameter 1. The greater value of these two endpoints was considered as the maximal single-parameter effect (Fig. 2A). Then:
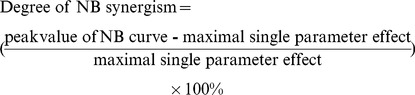
(16)


Based on Eq. 16, the degree of weak NB synergism (*i.e.*, NB curve is concave down and the peak response is obtained from either end point of NB curve) is always zero. Therefore, this degree was only calculated for parameter pairs that exhibit strong NB synergism, with degree >0. All other pairs that exhibit weak NB synergism were assigned a degree of 0. A negative degree corresponds to antagonism (*i.e.*, NB curve is concave up).

The degree of additive synergism was calculated using the maximal difference between the NB curve and AE curve, as follows (see also Fig. 2B).
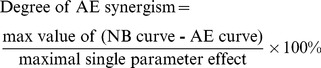
(17)A positive degree >1 corresponds to additive synergism, whereas a negative degree corresponds to antagonism.

Fourth-order Runge-Kutta integration was used for integration of ODEs, with a time step of 3 s. The model was programmed in XPP-Aut version 6.1 (http://www.math.pitt.edu/~bard/xpp/xpp.html). The XPP-Aut code is provided as Supplemental Material.

## Results

Perhaps the simplest model for studying synergism is a three-node model of two pathways that converge onto a single target. Here, each pathway represents stimulus-induced activation of an enzyme, which in turn modifies the activity or level of a target effector molecule. For generality, the activity of each pathway is simply represented as the level of an element (A or B). A and B converge to induce activation of the target, T (Fig. 1A1). To characterize the responses of this motif (and the additional motifs described below), a stimulus (*S*) is modeled as a brief (10 min) square-wave pulse, rising from a basal value of zero to an elevated value. To examine whether the strength of *S* affects synergism, the latter value is varied from weak (1) to strong (40) (non-dimensional units). After the pulse, *S* returns to zero. *S* concurrently activates A and B. The activities of A and B are dynamic variables *A* and *B* that follow first-order ODEs (see Model Development). Two variants of this model were analyzed. In Variant M, the effect of the two pathways on the expression of T is multiplicative. The activation rate of T is proportional to the product of the effects of A and B. Thus, Variant M is equivalent to a logical AND gate. In Variant A, the activation rate of T is proportional to the sum of the effects of *A* and B. Each element is thus able to separately activate T. Thus, Variant A is similar to a logical OR gate.

### Comparison of synergism in the multiplicative and additive model variants

**Figure 3:**
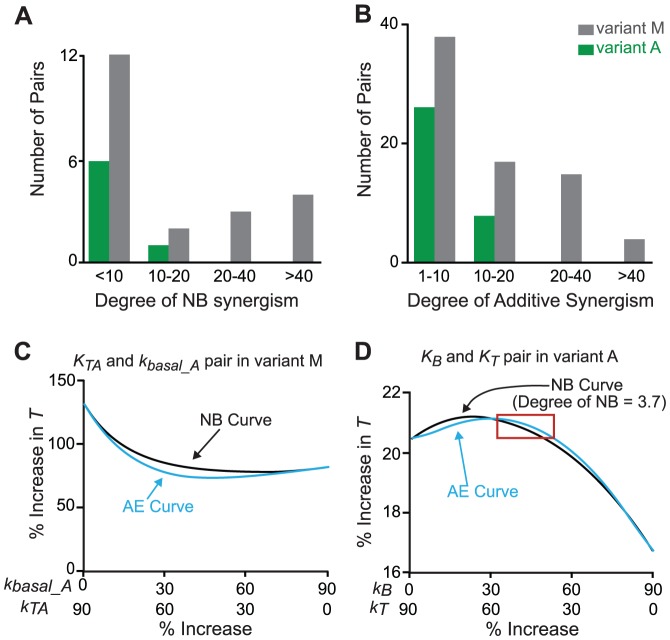
Histograms of NB synergism (A) and additive synergism (B) degrees from 91 parameter pairs for Variant M (grey bars) and Variant A (green bars) (*S* = 1). (**C**) Variation of the parameter pair *K_TA_*/*k_basal_A_* in Variant M shows that additive synergism can exist in the absence of NB synergism. *k_basal_A_* increases from 0 to 90% as *K_TA_* concurrently decreases from 90 to 0%. (**D**) Variation of *K_B_*/*K_T_* in Variant A shows that NB synergism can exist in the absence of additive synergism (red box, concave-down NB curve is below AE curve).

For Variants M and A, after systematic simulation of the effects of modifying 91 pairs of parameters, the degrees of NB and additive synergism for each parameter pair were evaluated (Fig. 3). Strong stimuli might induce a ceiling effect, which could preclude the synergistic effect of combined parameter changes. Therefore, in these simulations *S* = 1. In Variant M, 21 parameter pairs had a degree of NB synergism >0, and 74 pairs had a degree of additive synergism >1. In Variant A, only 7 parameter pairs had a degree of NB synergism >0, and 34 pairs had a degree of additive synergism >1. The histograms of the degrees of NB synergism and additive synergism for the 91 parameter pairs show that: 1) None of the parameter pairs in Variant A had a degree of NB synergism >20, whereas 7 parameter pairs in Variant M had a degree of NB synergism >20 (Fig. 3A), and 2) No parameter pairs in Variant A had a degree of additive synergism >20, whereas 19 parameter pairs in Variant M had a degree of additive synergism >20 (Fig. 3B). Thus the multiplicative effects of A and B on T in Variant M produced stronger NB and additive synergism than did the additive effects of A and B on T in Variant A.

In Variant M, all parameter pairs displaying NB synergism also produced additive synergism, whereas parameter pairs displaying additive synergism did not necessarily produce NB synergism. For example, with Variant M, the *k_basal_A_*/*K_TA_* pair produced antagonism in the NB curve (*i.e.*, the NB curve is concave up). However, this pair still produced additive synergism (Fig. 3C). In contrast, with Variant A, the *K_B_*/*K_T_* pair produced an AE curve that, in some portions, was above the NB curve, even though NB synergism was present (red box in Fig. 3D). Therefore, additive synergism may be observed in the absence of NB synergism, and NB synergism may be observed in the absence of additive synergism. When the AE curve is above the NB curve, the effect of the combined parameter variations (the NB curve) is less than the sum of the effects of the individual parameter variations (the AE curve). This corresponds to additive antagonism, and yields a negative degree (Eq. 17).

Because Variant M produced, on average, stronger NB and additive synergism than did Variant A, subsequent simulations were performed with Variant M.

### Parameter sensitivity analysis

**Figure 4:**
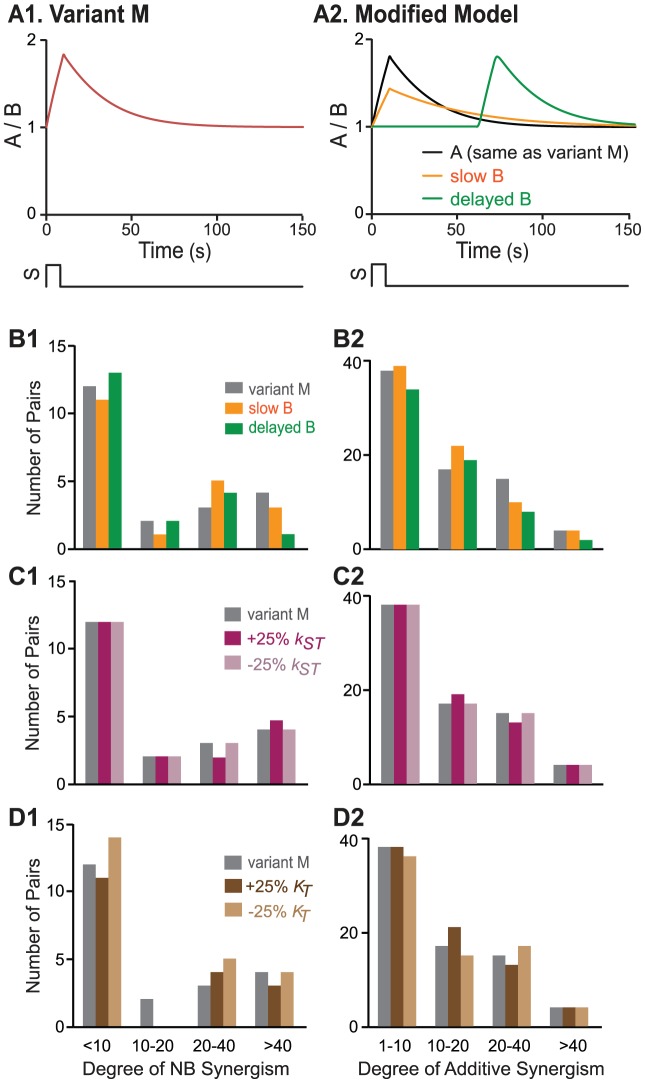
For Variant M (*S* = 1), the histograms of synergism degrees from 91 parameter pairs were compared between standard parameter values and varied parameter values. (**A**) The stimulus and response curves of A (see Eq. 1) and B (see Eq. 2) with standard parameter values (**A1**) and with parameters altered to give slower B dynamics (orange trace), or delayed B dynamics (green trace), with dynamics of A unchanged (black trace) (**A2**). (**B**) The histograms of NB synergism (**B1**) and additive synergism (**B2**) degrees with standard parameter values (grey bars) and with parameters altered to give slower B dynamics (orange bars) or delayed B dynamics (green bars). (**C**) The histograms of NB synergism (**C1**) and additive synergism (**C2**) degrees with standard parameter values (grey bars), with *k_ST_* increased by 25% (red bars), and with *k_ST_* reduced by 25% (pink bars). (**D**) The histograms of NB synergism (**D1**) and additive synergism (**D2**) degrees with standard values (grey bars), *K_T_* increased by 25% (dark brown bars), and *K_T_* reduced by 25% (light brown bars).

In Variant M, for simplicity, the standard parameter values of elements A and B were identical, thus the time courses of *A* and *B* following a stimulus *S* were identical (Fig. 4A1). To test whether a difference in the dynamics of these two elements would affect synergism, parameters for element B (*k_sB_*, *k_basal_B_*, and *k_dB_*) were all reduced by 50%. This modification made the activation and deactivation of B somewhat slower than that of A. Because of the relatively slower kinetics, the peak amplitude of *B* was reduced by ∼50% (Fig. 4A2), and the subsequent decay of *B* was slowed. NB and additive synergism were simulated after parameter modification. The histograms of NB and additive synergism degrees were compared between the models with fast B dynamics (original Variant M) and with slower B dynamics (Fig. 4B). Only slight shifts were observed in the distribution of NB and additive synergism degrees with slower B dynamics (Fig. 4B). In this case, similar to the original model Variant M, 9 parameter pairs had a degree of NB synergism >10, and 36 pairs had a degree of additive synergism >10 (Fig. 4B). In another test, the activation of B by *S* was delayed by 60 min compared to activation of A (Fig. 4A2), and the effect on synergism was assessed. With delayed B activation, 7 parameter pairs still had a degree of NB synergism >10, and 29 pairs had a degree of additive synergism >10 (Fig. 4B). Therefore, NB and additive synergism in Variant M were robust to moderate variations in dynamics.

In further simulations, the percent reduction of *k_sB_*, *k_basal_B_*, and *k_dB_* was increased to 70% and then to 90%, which made the activation and deactivation of B much slower than A. These changes led to a decrease in the number of parameter pairs showing synergism. When parameters for B were reduced by 70%, 7 parameter pairs had a degree of NB synergism >10 and none had a degree >40. 33 pairs had a degree of additive synergism >10 but none had a degree >40. When parameters for B were reduced by 90%, only 3 parameter pairs had a degree of NB synergism >10 and none had a degree >20. 28 pairs had a degree of additive synergism >10 but none had a degree >40. Although 50%, 70% and 90% were arbitrarily selected, they effectively represent the range of substantial variation of kinetics of B. These results suggest that the use of combined parameter changes might be less effective in inducing synergy if two converging pathways have very different dynamics. In the simulations, the highest degrees of synergism were produced when the basal parameter values governing the dynamics of both pathways were identical. Therefore, further analyses of effects of stimulus strength and network motifs on synergism were performed for the optimal initial condition (identical basal dynamics of elements A and B).

To further test the robustness of Variant M, parameter sensitivity analysis was performed for the dynamics of *T*. Six parameters that affect the dynamics of *T* (Eq. 3) were altered by either −25% or +25% from their standard values. NB and additive synergism for the 91 parameter pairs were simulated after each of these 12 parameter modifications. The histograms of NB and additive synergism degrees were compared for the cases of standard and varied parameters. Both NB and additive synergism were robust to these moderate variations in *T* dynamics. For example, after reducing or increasing *k_ST_* by 25% or reducing *K_T_* by 25%, 9 parameter pairs exhibited a degree of NB synergism >10, and 36 parameter pairs had a degree of additive synergism >10 (Figs. 4C–D). These pair numbers were the same as with standard parameter values. After increasing *K_T_* by 25%, 7 parameter pairs exhibited a degree of NB synergism >10, and 38 parameter pairs had a degree of additive synergism >10 (Fig. 4D).

Initially, the effects of stimulus strength and network motifs on synergism for all 91 parameter pairs were examined. However, the most dramatic changes in the dynamics of the motifs were related to alterations in the deactivation rate constants of elements A and B, *k_dA_* and *k_dB_*. Therefore, the detailed analyses of effects of stimulus strength and network motifs on synergism concentrated on variations to the three parameter pairs (*k_dA_*, *k_dB_*), (*k_dA_*, *k_ST_*), and (*k_dA_*, *K_T_*), each of which includes at least one of these deactivation rate constants. Each of these pairs consistently displayed substantially different degrees of synergism. For example, with *S* = 1, throughout almost all parameter variations, *k_dA_* and *k_dB_* maintained degrees of both NB and additive synergism >40, *k_dA_* and *k_ST_* maintained degrees of NB synergism >5 and additive synergism >20, and *k_dA_* and *K_T_* maintained a degree of additive synergism >10 and a degree of NB synergism = 0. For the *k_dA_*/*K_T_* pair the NB curve remained concave down with a maximal at an endpoint (weak nonlinear blending).

### The effects of stimulus strength on NB synergism and additive synergism

**Figure 5:**
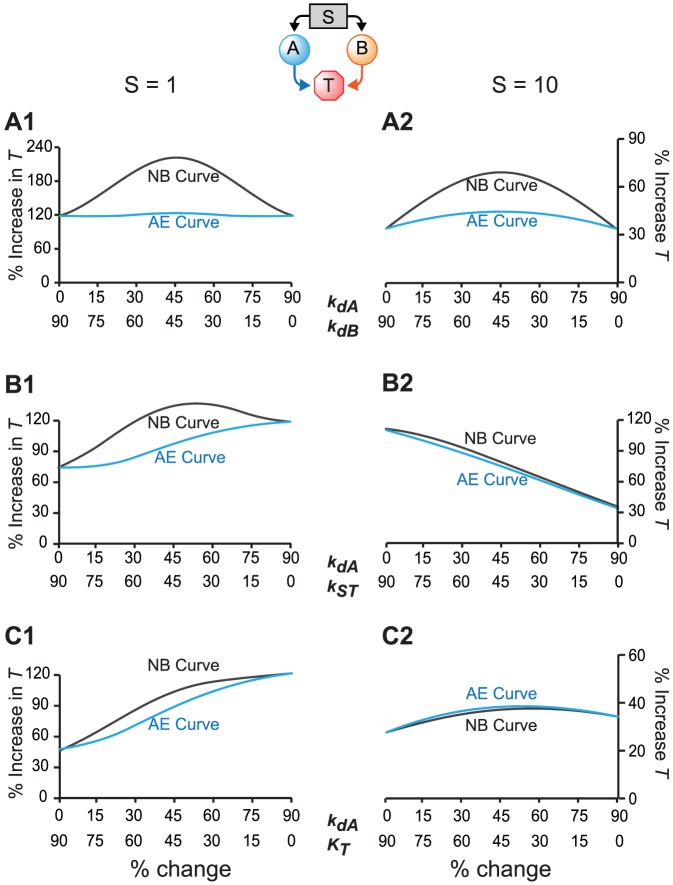
Simulations with Variant M generated NB curves and AE curves for three pairs of parameters, which show strong NB synergism (*k_dA_*/*k_dB_*) (A), moderate NB synergism (*k_dA_*/*k_ST_*) (B), and weak NB (*k_dA_*/*K_T_*) (C). *S* = 1 in the left column, and *S* = 10 in the right column.

Decreasing *k_dA_* or *k_dB_* prolongs activities of A or B. When a decrease in *k_dA_* was paired with a decrease in *k_dB_*, strong NB synergism resulted for *S* = 1 (Fig. 5A1). *k_ST_* is the activation rate of T induced by A and B. When a decrease in *k_dA_* was paired with an increase in *k_ST_*, moderate NB synergism resulted for *S* = 1 (Fig. 5B1). *K_T_* is the dissociation constant for deactivation of T. A decrease in *k_dA_* was paired with an increase in *K_T_*, which directly elevated the peak level of *T*. This pair of parameter changes produced no strong NB synergism (degree of 0), and yielded only weak additive synergism, when *S* = 1 (Fig. 5C1).

The AE curves of these pairs under different strengths of stimulus were simulated: *S* = 1 (Figs. 5A1, B1, C1) and *S* = 10 (Figs. 5A2, B2, C2). For *S* = 1, additive synergism is also evident for the *k_dA_*/*k_dB_* pair. The NB curve was well above the AE curve (Fig. 5A1). For the *k_dA_*/*k_ST_* pair, additive synergism was strong, with the NB curve lying significantly above the AE curve (Fig. 5B1). For *k_dA_*/*K_T_*, only slight additive synergism remained (Fig. 5C1). Interestingly *k_dA_*/*k_dB_*, which produced the strongest NB synergism, also produced the greatest increase of T (Figs. 5A1, B1, C1). This result indicates that the lack of strong NB synergism with the other two pairs is not due to saturation of the peak level of *T*.

When *S* = 10, for *k_dA_*/*k_dB_*, both NB synergism and additive synergism were still evident (Fig. 5A2). For *k_dA_*/*k_ST_*, the NB curve was only slightly above the AE curve, and NB synergism was almost absent (Fig. 5B2). For *k_dA_*/*K_T_*, additive synergism was reversed, because the AE curve was slightly above the NB curve (antagonism) (Fig. 5C2). Because *S* = 10 induced a higher control peak level of *T* than *S* = 1 in the absence of any parameter changes, the additional percentage increases of the *T* peak due to parameter variations were smaller when *S* = 10 (Figs. 5A2, B2, C2) than when *S* = 1 (Figs. 5A1, B1, C1), which indicated some saturation of the peak level of *T* for the higher stimulus, *S* = 10. However the *k_dA_*/*k_dB_* pair, which produced the strongest NB synergism, yielded a greater increase of *T* than did *k_dA_*/*K_T_* (Figs. 5A2, C2). This result indicates that the saturation of peak *T* is not the only reason for reduction of synergism.

**Figure 6:**
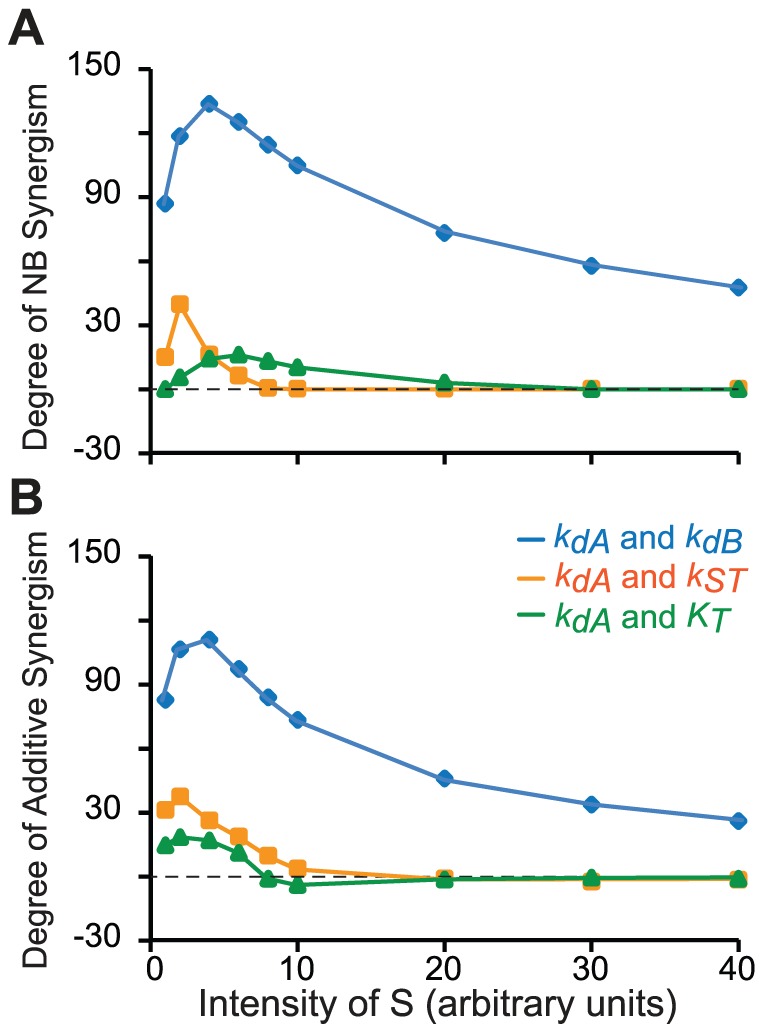
Simulations of three parameter pairs with Variant M generated dose-effect curves that describe the relationships between NB synergism and stimulus strength (A), and between additive synergism and stimulus strength (B). The strength of stimulus (*S*) was varied from 1 to 40.

The results of Fig. 5 indicate that increasing the strength of the stimulus tends to reduce NB synergism and additive synergism. To further test this hypothesis, the degrees of NB synergism and additive synergism were measured under a series of stimuli ranging from *S* = 1 to *S* = 40. Fig. 6 illustrates dose-effect curves of synergism *vs.* stimulus strength. Although, for all three pairs of parameters, NB synergism and additive synergism gradually decreased with increasing *S* when *S*>10, the dose-effect curves were not monotonic, displaying a type of inverted U-shaped curve. Each pair required a different stimulus strength to maximize each type of synergism. For each pair, maximal NB synergism and additive synergism could occur at distinct stimulus strengths.

### The effects of network motifs on NB synergism and additive synergism

#### Mutual excitation loop between A and B

The effects of mutual excitation on NB synergism and additive synergism (Fig. 1B2) were simulated with the same three parameter pairs as above in the presence of positive feedback, when *S* = 1 (Fig. S1, panels A1, B1, C1) or *S* = 10 (Fig. S1, panels A2, B2, C2). No strong NB synergism, or strong additive synergism, was exhibited for any of the pairs. For *k_dA_*/*k_dB_*, the AE curve was well above the NB curve, and the NB curve was concave up, illustrating antagonism (Fig. S1A1). For *k_dA_*/*k_ST_* (Fig. S1B1), and *k_dA_*/*K_T_* (Fig. S1C1), the NB and AE curves were close or intertwined. Compared to the corresponding AE and NB curves in the original Variant M (Fig. 5), it appears that NB synergism and additive synergism are both greatly reduced, for all three pairs, by the presence of this form of positive feedback. Indeed, the degrees of NB and additive synergism were <5 for all three pairs, whereas without excitation the degrees of NB synergism and additive synergism for *k_dA_*/*k_dB_* and *k_dA_*/*k_ST_* were >10, and the degree of additive synergism for *k_dA_*/*K_T_* was also >10.

When *S* = 10, the NB and AE curves were also close for all three pairs. Additive antagonism was nearly eliminated when *S* = 10. Mutual excitation also increased the peak level of *T*. This effect was particularly significant when *S* = 10, because the additional percentage increases of the *T* peak due to parameter variations were much smaller when *S* = 10 than when *S* = 1, which indicated saturation of the peak level of *T*. Therefore, with mutual excitation, increasing stimulus strength tends to eliminate both synergism and antagonism, apparently due to a ceiling effect caused by the combination of mutual excitation and strong stimulation.

#### Positive feedback from T to A and B

The effects of positive feedback from T to A and B (Fig. 1E) on NB synergism and additive synergism were similar to the effects of a mutual excitation loop between A and B (Fig. S5). Reduced synergism, or even antagonism, was evident when *S* = 1. Increasing the stimulus strength eliminated antagonism, possibly due to a ceiling effect.

#### Mutual inhibition loop between A and B

The effects of a mutual inhibition loop on NB synergism and additive synergism (Fig. 1B3) were simulated with the same three parameter pairs when *S* = 1 (Fig. S2, panels A1, B1, C1) or *S* = 10 (Fig. S2, panels A2, B2, C2). When *S* = 1, for *k_dA_*/*k_dB_*, both NB synergism and additive synergism were greatly enhanced by the inhibition (compare Figs. 5A1 and S2A1). The degrees of both forms of synergism were enhanced at least four-fold. In contrast, for *k_dA_*/*k_ST_* and *k_dA_*/*K_T_*, the NB and AE curves were intertwined. Compared to the corresponding AE and NB curves in the original Variant M (Fig. 5), NB synergism and additive synergism were greatly reduced.

The differential effects of mutual inhibition on synergism for different parameter pairs may be due to the fact that mutual inhibition and *k_dA_*/*k_dB_* are both involved in the deactivation of A and B, whereas *k_ST_* and *K_T_* are involved in the activation of T. Mutual inhibition enhanced NB synergism and additive synergism if both parameters were chosen from the reactions regulated by the inhibition. In contrast, mutual inhibition reduced both forms of synergism if at least one of the parameters was chosen from a different reaction or interaction, even if that reaction was downstream and indirectly regulated by the inhibition.

When *S* = 10, for *k_dA_*/*k_dB_*, both NB synergism and additive synergism remained strong. The NB curve was well above the AE curve (Fig. S2A2). The degrees of NB synergism and additive synergism decreased by ∼40% as compared to the degrees when *S* = 1. However, the presence of mutual inhibition still increased the values of both degrees by >100%. For *k_dA_*/*k_ST_* and *k_dA_*/*K_T_*, the NB and AE curves were no longer intertwined. The AE curves were slightly below the NB curves. In addition, *k_dA_*/*K_T_* shows substantial NB synergism when *S* = 10 (degree of NB synergism = 23). Thus for these two parameter pairs, with mutual inhibition, both forms of synergism are enhanced with increasing stimulus strength.

#### Positive auto-regulation of A

The effects of a positive auto-regulation loop on NB synergism and additive synergism (Fig. 1C1) were simulated with the same three parameter pairs when *S* = 1 (Fig. S3, panels A1, B1, C1) or *S* = 10 (Fig. S3, panels A2, B2, C2). When *S* = 1, for *k_dA_*/*k_dB_*, both NB synergism and additive synergism were strong. The NB curve was well above the AE curve (Fig. S3A1) and the degrees of both types of synergism were increased by at least 40% by positive auto-regulation. Indeed NB synergism was enhanced for all three pairs (compare Fig. 5, A1–C1 with Fig. S3, A1–C1). For *k_dA_*/*k_ST_* and *k_dA_*/*K_T_*, NB synergism was evident. Surprisingly, however, for these two pairs the AE curves were above the NB curves (additive antagonism). Additive synergism was reversed for *k_dA_*/*K_ST_* (Fig. S3B1) and *k_dA_*/*K_T_* (Fig. S3C1). Therefore for a weak stimulus (S = 1), positive auto-regulation enhances NB synergism for all pairs, but has diverse, parameter-specific effects on additive synergism. When *S* = 10, the NB and AE curves were close for all three pairs and no substantial NB synergism or additive synergism was observed. The positive auto-regulation eliminates NB synergism and additive synergism for a strong stimulus. Similarly to the mutual excitation loop discussed previously, this elimination of synergism for a strong stimulus might be due to an enhanced ceiling effect.

#### Negative auto-regulation of A

The effects of a negative auto-regulation loop on NB synergism and additive synergism (Fig. 1C2) were simulated in the presence of negative auto-regulation for the same parameter pairs. When *S* = 1, the degrees of NB synergism and additive synergism for all three pairs were at least 25% lower than the corresponding synergism degrees in the absence of the negative auto-regulation loop (the original Variant M). Therefore, negative auto-regulation reduces NB synergism and additive synergism when the stimulus is weak. For S = 10, negative auto-regulation enhanced NB synergism and additive synergism for *k_dA_*/*k_ST_*, and additive synergism for *k_dA_*/*K_T_*, compared to the corresponding degrees without negative auto-regulation. However, negative auto-regulation reduced NB synergism and additive synergism for *k_dA_*/*k_dB_*, and NB synergism for *k_dA_*/*K_T_*. The negative auto-regulation loop has a complicated, parameter-specific and stimulus-dependent effect on both forms of synergism.

#### Coherent positive feed-forward loop, or negative feed-forward loop, from A to B

For the same three parameter pairs, in the presence of a positive feed forward loop (*P-FFL*) (Fig. 1B1A), the degrees of additive synergism were negative or <1 when *S* = 10. When *S* = 1, for all pairs, these degrees of additive synergism were >50% less than the corresponding degrees in the absence of the feed-forward loop. The degrees of NB synergism for all three pairs were always 0, except for *k_dA_*/*k_ST_* when *S* = 10 (degree = 4). The feed forward loop reduces NB synergism and additive synergism.

The negative feed forward loop (*N-FFL*) (Fig. 1B1B) had similar effects as did the *P-FFL*. For the same three parameter pairs, the degrees of NB and additive synergism for all pairs were reduced regardless of stimuli.

#### Negative feedback between A and B, or from T to A and B

The effects of a negative feedback loop between A and B (Fig. 1D1) were simulated for the same parameter pairs when *S* = 1 and when *S* = 10. With this negative feedback, the degrees of NB synergism for all pairs were zero regardless of stimuli. When *S* = 1, for all pairs, the degrees of additive synergism were >50% less than the corresponding degrees in the absence of negative feedback. When *S* = 10, for *k_dA_*/*k_dB_*, the degree of additive synergism was ∼0.9. In contrast, the degree of additive synergism for *k_dA_*/*k_ST_* was ∼26, whereas the degree was ∼4 in the absence of negative feedback. The degree of additive synergism for *k_dA_*/*K_T_* was ∼7, whereas the degree was negative without negative feedback. This type of negative feedback reduces NB synergism and exhibits a parameter-specific and stimulus-dependent effect on additive synergism.

Finally, the effects of a negative feedback loop in which increased *T* accelerates deactivation of A and B (Fig. 1D2) were simulated when *S* = 1 (Fig. S4, panels A1, B1, C1) or *S* = 10 (Fig. S4, panels A2, B2, C2). When *S* = 1, the degrees of NB and additive synergism for *k_dA_*/*k_dB_* were ∼20% greater than in the absence of negative feedback, whereas the degrees of additive and NB synergism for *k_dA_*/*k_ST_* were, respectively, ∼40% and ∼5% lower. The degree of additive synergism for *k_dA_*/*K_T_* was reduced by ∼25%. In contrast, the degree of NB synergism for *k_dA_*/*K_T_* was small but positive (∼4), whereas the degree was 0 in the absence of negative feedback. Negative feedback from T to A and B has a diverse, parameter-specific effect on both forms of synergism.

When *S* = 10, NB and additive synergism was enhanced for all three pairs. For *k_dA_*/*k_dB_* the degrees of NB and additive synergism were at least 100% greater than in the absence of negative feedback from T to A and B. For *k_dA_*/*k_ST_* the degree of NB synergism was small (<1) but positive and the degree of additive synergism was ∼11, both increased above the degrees without feedback (NB synergism: 0; additive synergism: ∼4). For *k_dA_*/*K_T_* the degree of NB synergism was ∼22, more than 100% greater than without negative feedback (∼10). Additive synergism was also present (degree ∼11), in contrast to the antagonism seen without negative feedback (degree ∼−4) (Fig. 5B2). Negative feedback from T to A and B can enhance both NB and additive synergism when the stimulus is sufficiently strong.

#### Substantial enhancement of synergism was only seen for a subset of motifs

**Figure 7:**
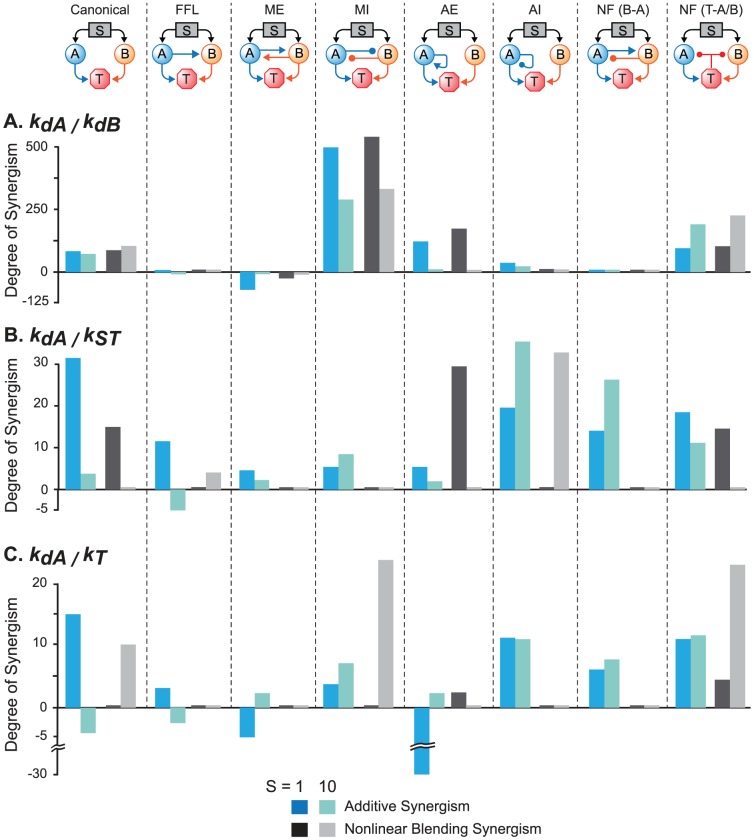
Summary of the degrees of NB and additive synergism observed for all motifs for *S* = 1 and *S* = 10. Degrees of synergism are plotted for *k_dA_*/*k_dB_* (**A**), *k_dA_/k_ST_* (**B**), and *k_dA_/K_T_* (**C**). Motif abbreviations are as in Fig. 1. Vertical dashed lines delineate each motif and its associated synergism degrees (four for each combination of a motif and a parameter pair). For each motif and parameter pair, the degrees of additive synergism are plotted as blue and light green bars, and the degrees of additive synergism are plotted as black and grey bars, for *S* = 1 and *S* = 10 respectively. Negative values represent degrees of antagonism. In a few cases, the NB and AE curves were intertwined (*e.g.*, Fig. S1B1) and exhibited both additive synergism and additive antagonism. For those cases only positive values (synergism) are plotted. The values of some data are too small to be easily visualized.

Fig. 7 summarizes the degrees of NB and additive synergism simulated for the canonical model and seven other motifs, for *S* = 1 and 10. Positive feedback from T to A and B, and the negative feed forward loop, were not included in Fig. 7, because their effects on synergism and antagonism were respectively similar to mutual excitation and to the positive feed forward loop. Variations of *k_dA_*/*k_dB_* in the mutual inhibition loop produced the greatest enhancement of synergism when compared to the canonical model. NB and additive synergism were greatly enhanced, and degrees in excess of 250 were simulated. However, with the other parameter pairs in the mutual inhibition loop, only *k_dA_*/*K_T_* showed an evident synergism increase, and only for NB synergism for *S* = 10. Negative feedback from T to A and B yielded a similar pattern. Substantial enhancement of all forms of synergism was again seen for *k_dA_*/*k_dB_*, especially for *S* = 10. Again, with the other parameter pairs, only *k_dA_*/*K_T_* exhibited substantially enhanced synergism, and only for NB synergism for S = 10. In both motifs, *k_dA_*/*k_ST_* and *k_dA_*/*K_T_* showed an evident but modest synergism increase for additive synergism for *S* = 10, although some degrees did not exceed 10 or were only slightly above 10.

For *k_dA_*/*k_dB_* in other motifs, positive auto-regulation was also seen to substantially enhance both forms of synergism, but only for *S* = 1, whereas for *S* = 10, both forms were abolished. For *k_dA_*/*k_ST_*, three other instances of substantial synergism enhancement (degrees >10) were seen in Fig. 7. Positive auto-regulation increased the NB synergism degree to 29 for *S* = 1. Negative auto-regulation enhanced both forms of synergism for *k_dA_*/*k_ST_*, but only for *S* = 10, and a negative feedback loop between A and B enhanced additive synergism for *k_dA_*/*k_ST_* for *S* = 10. The two remaining motifs, the mutual excitation loop and the positive feed forward loop, did not exhibit significant synergism for any conditions simulated.

Indeed, *k_dA_*/*k_dB_* in the mutual excitation loop exhibited evident NB and additive antagonism. A degree as negative as −70 for additive synergism was simulated for *S* = 1. One other instance of substantial antagonism (degree ∼−28) occurred for *k_dA_*/*K_T_* and positive auto-regulation (additive antagonism, *S* = 1).

### The effects of network motifs on NB synergism and additive synergism in the CREB model

**Figure 8:**
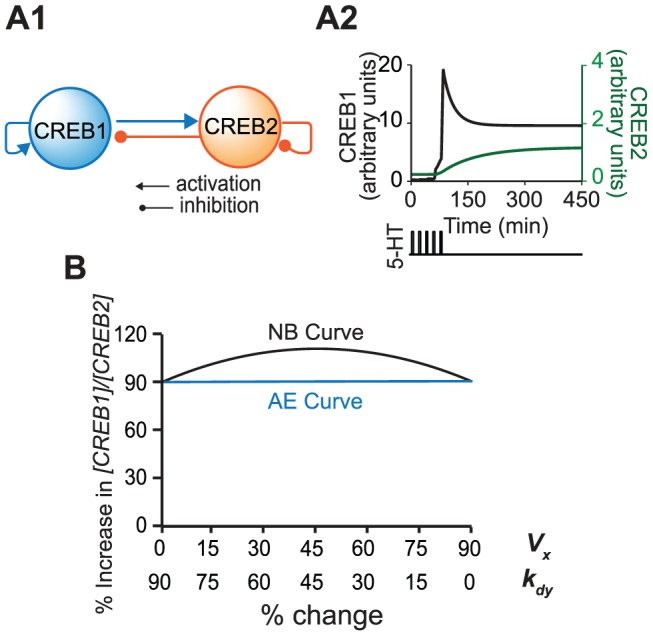
The model of Song et al. [26] and simulations. (**A1**) The feedback loops described by the model. (**A2**) After 5 pulses of 5-HT treatment, CREB1 and CREB2 switch from a LOW state to a HIGH state. (**B**) The NB curve and AE curve for the parameter pair *V_x_*/*k_dy_*. This pair shows strong NB synergism, and additive synergism.

Three network motifs are included in the model of Song et al. (2007) (Fig. 8A1): a positive auto-regulatory loop in which CREB1 enhances its own synthesis; a negative auto-regulatory loop in which CREB2 represses its own synthesis; and a negative feedback loop in which CREB2 represses the synthesis of CREB1. There are 8 parameters in Eqs. 14 and 15, leading to 28 possible distinct combinations of two parameters. The effects of these three motifs on NB synergism and additive synergism were examined by varying all 28 parameter pairs.

In Song et al. [26], when the standard parameter values are used, the model has two stable steady states: LOW and HIGH states. *V_x_* is transiently increased from 0.4 min^−1^ to 3.7 min^−1^ to simulate the application of a neurotransmitter, 5-HT [26]. A standard 5-HT protocol that is widely used to induce long-term synaptic facilitation (LTF) (5 pulses of 5 min 5-HT with interstimulus interval of 20 min) [39] is simulated, by increasing *V_x_* for 5 min for each of the 5 pulses. After the 5-HT stimulus, CREB1 and CREB2 were switched from the LOW state to the HIGH state (Fig. 8A2). The ratio of [CREB1]/[CREB2] after they converge to the HIGH state was considered to represent the response to 5-HT. An increase in this ratio corresponds to an increase in the level of the transcription activator CREB1 and/or a decrease in the level of the transcription repressor CREB2. Therefore an increase in this ratio corresponds to increased induction of genes necessary for LTF. LTF is a cellular correlate of long-term memory (LTM), and an increase of [CREB1]/[CREB2] corresponds qualitatively to improved formation of some forms of LTM. The [CREB1]/[CREB2] ratio in the absence of parameter changes was regarded as the control, and the percentage increase of the ratio over the control was taken as the simulated response to each of the 28 variations in parameter pairs. As was done previously for the three-node motifs (Methods), for each parameter pair, individual parameters were varied in the direction that acted to increase the response, *i.e.*, the [CREB1]/[CREB2] ratio.

After systematic, concurrent variation of each of the 28 pairs of parameters, only one pair of parameters, *V_x_*/*K_dy_*, was found to exhibit degrees of NB and additive synergism that exceeded 20 (Fig. 8B). *V_x_* is the maximum induced synthesis rate of CREB1, whereas *K_dy_* is the degradation rate of CREB2. In these simulations, *V_x_* and *K_dy_* were both increased, because for both parameters, an increase acts to elevate [CREB1]/[CREB2]. Increasing *V_x_* enhances the strength of the positive auto-regulatory loop of CREB1. Increasing *K_dy_* suppresses the negative feedback between CREB1 and CREB2 and suppresses the negative auto-regulatory loop of CREB2 by accelerating the degradation of CREB2. Thus increasing positive auto-regulation while simultaneously suppressing negative auto-regulation was found to be the only way to obtain substantial NB synergism and additive synergism. These simulation results are consistent with the earlier results from the three-node motifs, that positive auto-regulation acts to enhance NB synergism, and that negative auto-regulation acts to suppress synergism.

## Discussion

The effects of varying parameter combinations in the context of common motifs in biochemical pathways were simulated, and for each motif, parameter combinations were identified that have a relatively greater likelihood of exhibiting synergism. For every motif (Fig. 1), simulations quantified degrees of NB synergism and of additive synergism for an extensive set of 91 parameter pairs. Three of these pairs, which each exhibited substantially different amounts of synergism, were then used to analyze parameter combination effects in detail. By concurrent simulation of NB and AE curves, we were able to visualize which pairs produced synergism (e.g., Fig. 5A), and which produced antagonism (*e.g.*, Fig. 3C). Simulations demonstrated that NB synergism can exist without additive synergism, and vice versa (Figs. 3C, 3D).

### A canonical model that exhibits synergism

For the basic motif of two converging signaling pathways (Fig. 1A1), the model variant with a multiplicative effect of the pathways on the regulation of a target (Variant M) produced stronger synergism than did the model with a simply additive effect of the two pathways (Variant A). Thus, combined parameter variations may be less efficacious if they occur within signaling pathways that have additive effects on a downstream target. However, and as discussed below, a multiplicative mechanism does not guarantee synergistic effects in other motifs.

The synergism produced by Variant M was robust to modest variations of parameters governing the dynamics of signal pathways. In contrast, synergism was much more sensitive to the strength of stimulus and to the presence of feedback or feed forward interactions between elements and their target. NB and additive synergism were decreased when the stimulus became strong (Fig. 6). These results are not surprising, because increasing stimulus intensity will saturate the signaling cascade. This ceiling effect will prevent the combined parameter changes from further enhancing the activation of their target. However, the relationships between synergism and stimulus strength were non-monotonic (Fig. 6). Each parameter pair exhibited maximal synergism at a different stimulus strength. For example, *k_dA_*/*k_dB_* produced maximal NB synergism when *S* = 4, but *k_dA_*/*k_ST_* produced maximal NB synergism when *S* = 2 (Fig. 6). When *S* = 8, a simulated parameter combination was still effective (*i.e.*, synergism was observed) for *k_dA_*/*k_dB_*, but not for *k_dA_*/*k_ST_* (Fig. 6). These simulations suggest that empirically, if the physiological stimulus strength activating a given pathway varies, the optimal choice of combined drug therapy to target that pathway might also change.

### The effectiveness of paired parameter variations depends on the network motifs

Mutual excitation and mutual inhibition feedback loops are two motifs commonly observed in signaling pathways [29]. Variation of a pair of parameters yields synergistic effects in mutual inhibition motifs (Figs. 1B3, 7, S2), but has opposite effects on motifs with a mutual excitation loop (Fig. 1B2), where both NB and additive synergism (Figs. 7, S1) were eliminated. This elimination may be due to the self-reinforcing effect of the positive feedback, enhancing the ability of combined parameter changes to saturate the response. Similar results were obtained for paired parameter changes in a positive feed forward motif (Fig. 1B1). These results suggest that it may not be advantageous to design a combined drug therapy for which both drugs activate a positive feedback loop similar to that of Fig. 1B2, or a feed forward loop similar to Fig. 1B1. In contrast, mutual inhibition tends to prevent activation of the target from saturating, and this effect may explain the enhanced synergism. Thus, a promising strategy for combination therapy might be to target drugs to elements of mutual inhibition loops.

The effects of other network motifs on synergism were also examined. A positive auto-regulation loop (Fig. 1C1) enhanced NB synergism only if: 1) the upstream stimulus activating the signaling pathway was weak, and 2) one or both of the parameters were part of the positive auto-regulation loop (Figs. 7, S3). Although positive auto-regulation surprisingly reversed additive synergism for some parameter pairs (Fig. S3), these simulations nevertheless suggest that given conditions 1) and 2), a positive auto-regulation loop may be a worthwhile target of a combination drug therapy. In contrast, a negative auto-regulation loop had a diverse, pair-specific effect on synergism (Fig. 7). When the upstream stimulus was weak, this auto-regulation tended to reduce synergism. Only for *k_dA_*/*k_ST_* and for a strong upstream stimulus was a substantial enhancement (degree >10) seen. These simulations suggest that a signal pathway with negative auto-regulation may not be a good target for combined drug therapies that affect pathway parameters in a similar manner to that modeled here. Simulations with the more biologically realistic model of [26], which contains multiple motifs, supported the above suggestions. With this model, the only way to obtain substantial NB synergism and additive synergism was to suppress negative auto-regulation while substantially enhancing positive auto-regulation.

The effects of a negative feedback loop between the target and an upstream element were similar to those of a mutual inhibition loop between elements (Figs. 7, S2, S4). In the negative feedback and the mutual inhibition loops:

- For *k_dA_*/*k_dB_*, both NB synergism and additive synergism were enhanced regardless of stimulus strength, compared to the model without feedback or inhibition.
- For *k_dA_*/*k_ST_*, additive synergism was enhanced when the stimulus was sufficiently strong (*S* = 10).
- For *k_dA_*/*K_T_*, both forms of synergism were enhanced when the stimulus was sufficiently strong (*S* = 10).

These results further support the suggestion that some pathways with negative interactions, either mutual inhibition between elements, or inhibition between a downstream target and an upstream element, may be good targets for combined drug therapy, especially when both drugs target the parameters involved in the negative interactions. However, a simple negative feedback loop between A and B (Fig. 1D1) may not be a good target for combined drug therapies that alter parameters in a way similar to that modeled here, because with this negative feedback, the degrees of NB synergism for all three parameter pairs were zero regardless of stimuli (Fig. 7).

Although elements A and B are representative of enzymes acting on a common target (T), the motifs of Figs. 1A1–1D2 are found in signaling pathways that also include regulation of gene expression [1], [29], [40]. Thus A, B, and/or T could alternatively refer to genes, or A and B could refer to transcription factors inducing gene T. Drug combinations targeting elements of motifs with such elements might include antisense RNA or siRNA to affect translation of a regulatory protein, or compounds that affect the activity of transcription factors.

Indeed, numerous feedback loops and feed-forward motifs have been identified in signaling pathways that include gene regulation and that are associated with cancer and other disorders. Cui *et al.* [4] mapped interactions among ∼1,600 genes associated with oncogenesis. Over 850 three-node FFLs similar to that in Fig. 1B1 were identified, and ∼200 feedback loops similar to those in Figs. 1B2 and 1B3 were found. One well-known example is the p53-mdm2 negative feedback loop [41]. Aberrant activation of the Ras→Raf→MAP kinase pathway is also commonly implicated in cancer [42], and a strong negative feedback loop within which activation of MAP kinase leads to inhibition of upstream Raf kinase has been identified [43]. Considerable effort is also being directed toward developing pharmacotherapies to improve learning and memory in individuals with cognitive deficits due to molecular lesions (*e.g.*, Rubinstein-Taybi syndrome [44]; or neurofibromatosis type 1 [45]). Long-term synaptic potentiation (LTP) is a correlate of learning and memory, and within signaling pathways important for LTP, numerous three-node FFLs and feedback loops have been identified [46]. Interestingly, over the entire human gene network, three-node FFLs are the most common regulatory motif [5].

### Sensitivity of synergism to initial parameter values

A series of sensitivity analyses were performed. The results indicated that although synergism and antagonism could be dependent on initial setting of individual parameter space, synergism may be less likely if parameters are concurrently changed in two converging pathways with substantially different dynamics. In the simulations, the highest degrees of synergism were produced when the basal parameter values governing the dynamics of two converging pathways were identical. This finding that choosing two target pathways with similar dynamics tends to favor synergism might be useful in designing some combination drug therapies.

### Implications for combination drug therapies

Combination drug therapies are commonly used for complex diseases and neurological disorders such as Alzheimer's disease, depression, traumatic brain injury, cancer, type 2 diabetes, and infections [47]–[54]. A potential benefit of combination therapies is synergism [50], [55]–[56]. With synergism, drugs administered together have a greater effect than would be predicted by simple addition of single-drug effects (*i.e.*, super-additive effects). Thus synergism allows for lower drug doses, minimizing undesirable effects. However, the molecular mechanisms underlying the synergism produced by combined drugs are not well understood for many combination drug therapies. In Axelrod et al. [57], 420 drug combinations were screened in 14 different cell lines. 84 combinations were found to generate synergism in multiple cell lines. The mechanistic analysis did help to suggest possible mechanisms involved in the induction of synergism. For example, the analysis implied that a combination of Ro31-8220 and lapatinib might produce synergism by their compensatory crosstalk between the p70S6 kinase and EGF receptor pathways [57]. However, none of the 84 combinations were synergistic in more than half of the 14 cell lines, and no pattern of lineage specificity was observed. Moreover, the authors found that even compounds from the same family or with similar structures cannot substitute for each other to induce synergism, which reflects the diversity of complex signaling networks.

In the current study, concurrent variation of parameter pairs to increase a response can represent the effect of a pair of drugs targeted to affect the corresponding interactions in a specific pathway motif. Therefore, alterations in parameter pairs that result in both NB synergism and additive synergism, and for which both types of synergism are robust to moderate variations in other parameters, may help to suggest, or to prioritize, drug combinations for empirical investigation.

This study did not focus on examining how motifs affect a parameter pair for varying initial values of all model parameters. Instead, the aim was to investigate how network motifs affect the degrees of synergism associated with variation of different types of parameter pairs, when the initial values of model parameters favor the induction of synergism. In the analysis of effects of stimulus strength and network motifs on synergism, we examined whether the motifs enhanced or eliminated the synergism for the parameter pairs that already exhibited synergism in the canonical model (*k_dA_*/*k_dB_* and *k_dA_*/*k_ST_*), and whether the motifs helped to generate synergism for the parameter pair that did not exhibit synergism in the canonical model (*k_dA_*/*K_T_*).

The network motifs examined in the present study are obvious simplifications of the processes taking place in realistic biological networks and the analysis of these motifs is not expected to yield a complete understanding of these networks and the ways in which combined parameter variations affect them. However, models of simplified motifs have their own advantages. They have relatively few parameters and a relatively simple mathematical structure, so it is possible to simulate and analyze the dynamics characteristic of a specific motif, which can be otherwise obscured within a complex network. Moreover, once the key dynamical elements of basic motifs are characterized, it is easier to incorporate these elements into more complicated and biologically realistic models. Under some circumstances, the dynamics characteristic of a single motif may dominate a local biological network [29], [58]. Indeed, Tyson and Novak [58] review several cases for which a single motif appears to dominate the dynamics of a cellular response. In these circumstances, the analysis presented here could be particularly useful for understanding cellular responses to pairs of drugs affecting that motif.

In summary, models such as those studied here provide insights into the dynamical properties of network motifs. Moreover, the stimulus protocols and parameter manipulations used here may help to predict the extent of synergism and consequently may prove useful in prioritizing empirical investigations of combination therapies. If combined drugs target signaling pathways that contain mutual excitation or positive feed forward interactions, a single drug might be as efficient as combined drugs. Whereas, if combined drugs target signaling pathways that contain a negative feedback loop between the target and an upstream element, or a mutual inhibition loop between elements, the combination may be more likely to exhibit empirical synergism. The usefulness of this approach is dependent on knowledge of the motifs that participate in signaling pathways affected by specific disorders. Often this information is currently limited. However, as progress is made in understanding motifs affected by diseases, the use of such computational models in the initial stage of designing combination drug therapies may become a common and efficient methodology.

## Supporting Information

Code S1 This file contains code written for the free, publicly available software package XPPAut. This code served as the basis for all the simulations in the manuscript.
(TXT)

Figure S1 Simulations in the model with a mutual excitation loop between A and B generated NB curves and AE curves for *k_dA_*/*k_dB_* (**A**), *k_dA_*/*k_ST_* (**B**) and *k_dA_*/*K_T_* (**C**). Stimulus was weak in the left column (*S* = 1), and strong in the right column (*S* = 10).
(EPS)

Figure S2 Simulations in the model with a mutual inhibition loop between A and B generated NB curves and AE curves for *k_dA_*/*k_dB_* (**A**), *k_dA_*/*k_ST_* (**B**) and *k_dA_*/*K_T_* (**C**). Stimulus was weak in the left column (*S* = 1), and strong in the right column (*S* = 10).
(EPS)

Figure S3 Simulations in the model with a positive auto-regulation loop of A generated NB curves and AE curves for *k_dA_*/*k_dB_* (**A**), *k_dA_*/*k_ST_* (**B**) and *k_dA_*/*K_T_* (**C**). Stimulus was weak in the left column (*S* = 1), and strong in the right column (*S* = 10).
(EPS)

Figure S4 Simulations in the model with negative feedback from T to A and B generated NB curves and AE curves for *k_dA_*/*k_dB_* (**A**), *k_dA_*/*k_ST_* (**B**) and *k_dA_*/*K_T_* (**C**). Stimulus was weak in the left column (*S* = 1), and strong in the right column (*S* = 10).
(EPS)

Figure S5 Simulations in the model with positive feedback from T to A and B generated NB curves and AE curves for *k_dA_*/*k_dB_* (**A**), *k_dA_*/*k_ST_* (**B**) and *k_dA_*/*K_T_* (**C**). Stimulus was weak in the left column (*S* = 1), and strong in the right column (*S* = 10).
(EPS)
